# Neuromuscular junctions are stable in patients with cancer cachexia

**DOI:** 10.1172/JCI128411

**Published:** 2020-02-10

**Authors:** Ines Boehm, Janice Miller, Thomas M. Wishart, Stephen J. Wigmore, Richard J.E. Skipworth, Ross A. Jones, Thomas H. Gillingwater

**Affiliations:** 1 Biomedical Sciences, Edinburgh Medical School, Edinburgh, United Kingdom.; 2 Clinical Surgery, Edinburgh Medical School and Royal Infirmary of Edinburgh, Edinburgh, United Kingdom.; 3 Roslin Institute, University of Edinburgh, Easter Bush Campus, Midlothian, United Kingdom.

**Keywords:** Neuroscience, Oncology, Neuromuscular disease

## Abstract

Cancer cachexia is a major cause of patient morbidity and mortality, with no efficacious treatment or management strategy. Despite cachexia sharing pathophysiological features with a number of neuromuscular wasting conditions, including age-related sarcopenia, the mechanisms underlying cachexia remain poorly understood. Studies of related conditions suggest that pathological targeting of the neuromuscular junction (NMJ) may play a key role in cachexia, but this has yet to be investigated in human patients. Here, high-resolution morphological analyses were undertaken on NMJs of rectus abdominis obtained from patients undergoing upper GI cancer surgery compared with controls (*N* = 30; *n* = 1,165 NMJs). Cancer patients included those with cachexia and weight-stable disease. Despite the low skeletal muscle index and significant muscle fiber atrophy (*P* < 0.0001) in patients with cachexia, NMJ morphology was fully conserved. No significant differences were observed in any of the pre- and postsynaptic variables measured. We conclude that NMJs remain structurally intact in rectus abdominis in both cancer and cachexia, suggesting that denervation of skeletal muscle is not a major driver of pathogenesis. The absence of NMJ pathology is in stark contrast to what is found in related conditions, such as age-related sarcopenia, and supports the hypothesis that intrinsic changes within skeletal muscle, independent of any changes in motor neurons, represent the primary locus of neuromuscular pathology in cancer cachexia.

## Graphical abstract

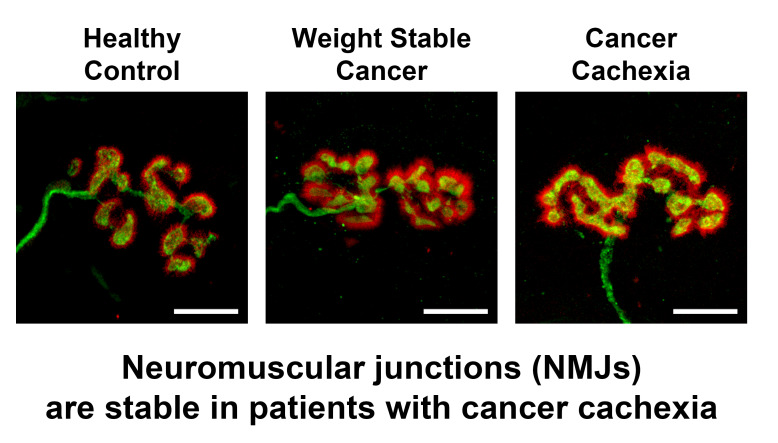

## Introduction

Cachexia is a severe and debilitating syndrome, commonly associated with cancer and characterized by the loss of muscle with or without corresponding loss of adipose tissue (1). Cancer cachexia is a major burden for both patients and health care systems globally, with profoundly negative impacts on the response to treatment, quality of life, and long-term survival of patients (2). At present, the consensus definition of cancer cachexia confirms loss of skeletal muscle as a key feature of the condition (3), largely mediated by proinflammatory cytokines and tumor-associated mediators, resulting in the activation of catabolic pathways in skeletal muscle (4). In this regard, cancer cachexia shares many of the muscle-specific and systemic inflammatory pathways common to the muscular dystrophies (5, 6).

Although the majority of research to date has focused on muscle abnormalities as the major locus of pathophysiology in cachexia, many lines of evidence have implicated the neuromuscular junction (NMJ) as a critical and early mediator of neuromuscular dysfunction and breakdown. Principally, NMJ dysfunction and denervation represent a shared hallmark of several related muscle-wasting conditions and neuromuscular diseases (7–9). For example, NMJ pathology is considered to represent a key early driver of neuromuscular defects in age-related sarcopenia (10–12), at least in part the result of an age-related loss of motor neurons (13). As cancer cachexia and sarcopenia share similar molecular mechanisms (14), and as cachexia is considered to be a multifactorial syndrome that includes components of both age-related sarcopenia and bed rest/reduced physical activity (15), the NMJ has similarly been implicated in the pathogenesis of cachexia. The identification of displaced mononuclei in muscle of cachectic patients and C26 tumor-bearing mice has been used to suggest the presence of denervation (16). Furthermore, a member of the ubiquitin-proteasome pathway (MuRF1) pivotal to muscle wasting in tumor-bearing mice (and other murine models of muscle wasting) is critical for maintenance of the NMJ (17). Additionally, recent mouse studies of mTOR signaling, a key regulator of protein synthesis that is suppressed by inflammatory mediators in cancer cachexia (18), have shown that muscle-specific deletion of mTOR or Raptor results in muscle fibrillation and NMJ fragmentation (19).

The role of the nerve-muscle interface in human cachexia is therefore of interest to both clinician and basic scientists and relevant to our understanding of disease pathophysiology and the development of effective treatments. Thus, cancer cachexia has been the subject of coculture/informatics projects (20) and ongoing patient studies. Experimental evidence supporting a direct role for NMJs in human cachexia is, however, highly reliant on studies of animal models of related conditions (21–23). Importantly, recent data have revealed striking and unexpected differences between the cellular and molecular anatomy of human NMJs compared with those of other model organisms (24), suggesting that findings from animal models may not be directly applicable to human patients.

The present study builds upon our recent work establishing a robust protocol to facilitate the sampling and high-resolution, quantitative morphological analyses of human NMJs from patients undergoing surgery (24, 25). We have adapted these protocols to facilitate a comprehensive analysis of the NMJ in patients with cancer cachexia.

## Results and Discussion

To investigate the role of the NMJ in human cancer cachexia, we performed a comprehensive morphometric analysis of the NMJ in samples of rectus abdominis (RA) muscle obtained from patients undergoing surgery for upper gastrointestinal (GI) cancer (Supplemental Table 1; supplemental material available online with this article; https://doi.org/10.1172/JCI128411DS1). RA was selected for 2 reasons: (a) it is readily accessible in the majority of surgical approaches to the abdomen and therefore a well-utilized muscle for sampling and characterization of human cancer cachexia (26) and (b) nerve roots innervating RA are unlikely to be affected by radiculopathy or other common spinal pathology, rendering neurogenic remodeling an unlikely possibility.

Cachectic patients demonstrated significantly lower skeletal muscle index (SMI) by computerized tomography (CT) (Supplemental Figure 1) criteria compared with weight stable patients (Supplemental Table 1) and also demonstrated a trend toward lower subcutaneous adiposity and higher visceral adiposity. These 2 body composition phenomena are associated with worsened outcomes in cancer patients (27–29), further confirming cachectic patients as a high-risk group. Two patients in the weight-stable cancer group exhibited a small degree of weight loss, but were not cachectic by the consensus definition (3, 30).

**Figure 1:**
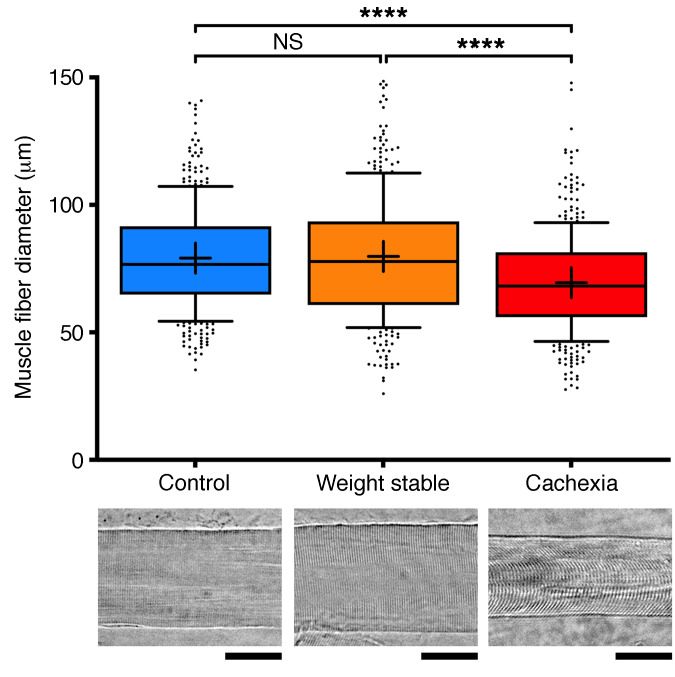
Atrophy of skeletal muscle fibers in cancer cachexia. Upper panel shows a box and whisker plot of muscle fiber diameters in control (*n* = 10 patients), weight-stable (*n* = 10), and cachectic (*n* = 10) patients. Bottom panels show representative micrographs of single, teased muscle fibers from control (left), weight-stable (middle), and cachectic (right) patients. Scale bars: 50 μm. Cachectic patients had significantly reduced muscle fiber diameters compared with weight-stable and control cases (control, *n* = 388; weight stable, *n* = 362; cachexia, *n*= 400 muscle fibers). Boxes contain the mean (+) and median (line) muscle-fiber diameters for the group and enclose the central 25th-75th percentile of the data, and whiskers extend from the 10th-90th percentile. Outlying data points are shown beyond the whiskers. ****P < 0.0001, 1-way ANOVA paired with Tukey’s post-hoc test. Individual *P* values are shown in Supplemental Table 2.

To confirm/validate the patient groupings based on the clinical and radiological guidelines (percentage of weight loss and SMI, respectively), we assessed muscle fiber diameter on teased muscle fiber preparations from RA. Mean muscle fiber diameter was significantly reduced (by almost 15%) in the cachectic patients compared with both control and weight-stable groups (*P* < 0.0001; Figure 1). However, there was no significant difference in mean muscle fiber diameter between control patients and those with weight-stable disease (*P* > 0.05; Figure 1). These observations are in keeping with published data showing marked muscle fiber atrophy in cachexia (31, 32) and support the patient group allocations based on consensus definition (3).

**Figure 2:**
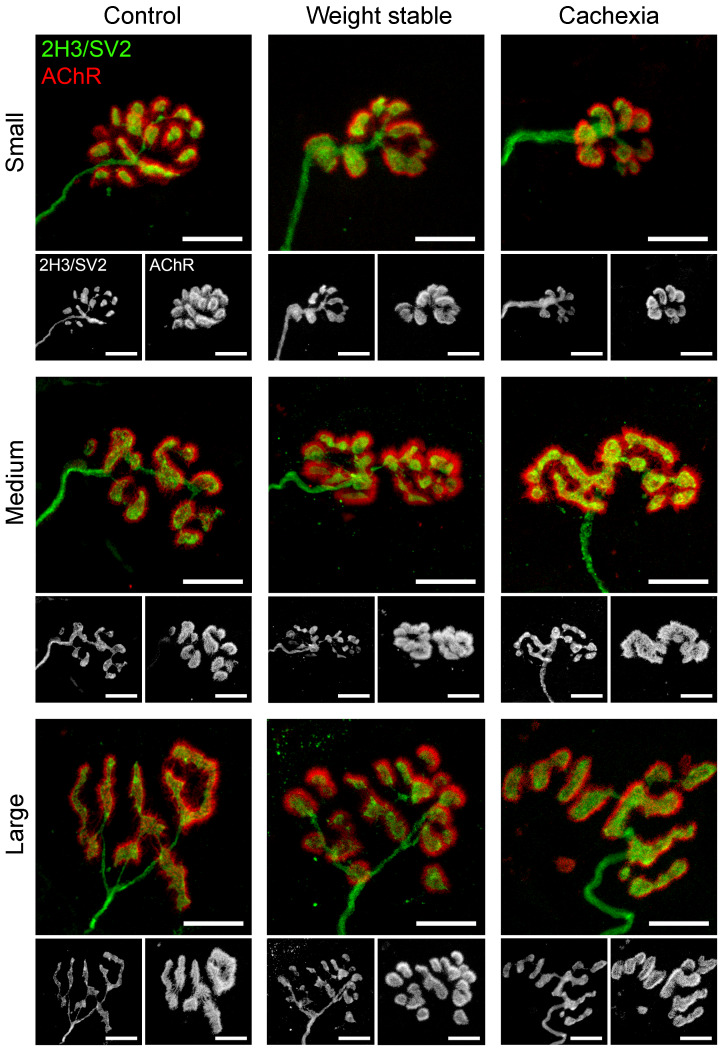
Conservation of NMJ morphology in cancer cachexia. Confocal micrographs of representative small, medium, and large NMJs from RA in the 3 patient groups. Despite heterogeneity in size and shape of individual NMJs, overall morphology was conserved across all groups, with no evidence of NMJ pathology in either the cachexia or weight-stable groups. Axon and nerve terminals are shown in green (2H3/SV2) and AChRs of the motor endplate in red (α-BTX). Scale bars: 10 μm.

**Figure 3:**
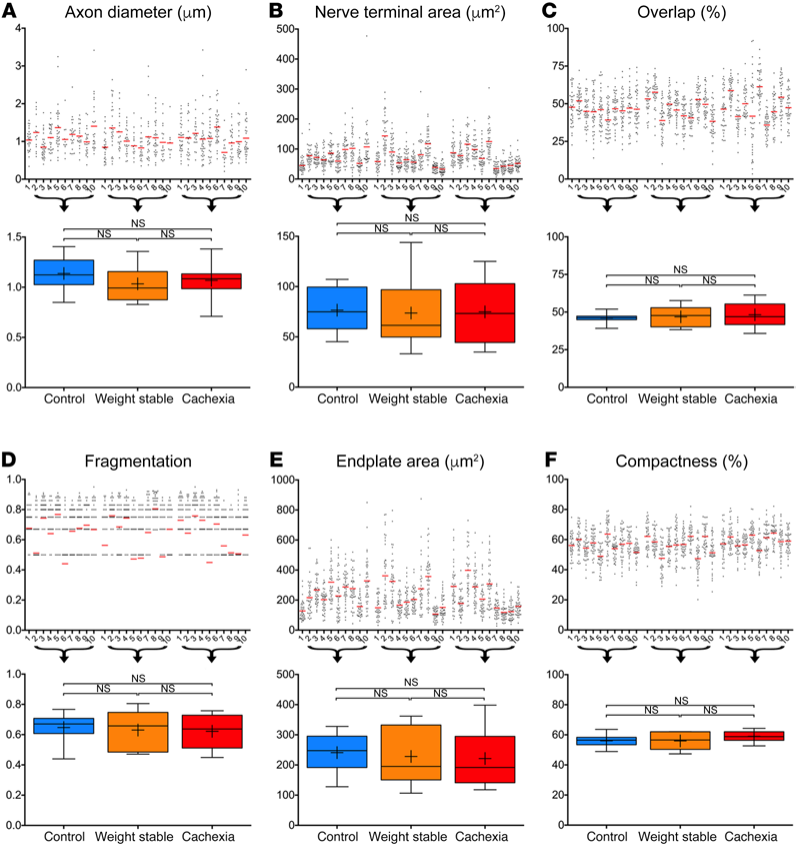
Structural integrity of the NMJ in cancer cachexia. Morphometric analysis using NMJ-morph revealed that NMJ morphology is conserved in both cachexia and weight-stable disease. Data presented as a pair of charts (scatterplot, above; box and whisker plot, below) for key NMJ variables, including measurements of axon diameter (**A**) and pre- and postsynaptic architecture (**B–F**). Scatterplots depict the approximately 40 individual NMJs (data points) for the 10 patients (1 to 10) in each group; the mean NMJ value is given by the red line; the observed heterogeneity is a normal feature of human NMJ morphology. Box and whisker plots constructed using the mean patient data in each group (10 patients; control NMJs, *n* = 387; weight stable NMJs, *n* = 386; cachexia NMJs, *n* = 392). Boxes contain the mean (+) and median (line) values for each NMJ variable and enclose the central 25th-75th percentile of the data; whiskers represent the maximum and minimum values. One-way ANOVA paired with Tukey’s post hoc test.

Given that muscle fiber atrophy would be predicted to result from and/or lead to NMJ instability based on findings from animal studies (8, 9), we next performed an initial qualitative assessment of NMJs. Despite the presence of muscle fiber atrophy in cachectic patients, NMJ morphology was indistinguishable from that observed in weight stable and control patients (Figure 2). NMJs of all 3 cohorts were noted to display the typical “nummular” morphology characteristic of human NMJs (24) and, despite the predicted heterogeneity in form across the complete pool of NMJs (Figure 3), we found no evidence of gross pathological changes or denervation of skeletal muscle fibers.

Although initial qualitative observations suggested an absence of gross pathology at the NMJ, more subtle changes in NMJ morphology could still have been present in the cachectic patients. We therefore undertook a comprehensive NMJ-morph analysis of all patient NMJs (Figure 3 and Supplemental Table 2). NMJs from RA were initially compared with our existing database of human NMJs from several lower limb muscles (24) to determine their likeness (or otherwise) to established human NMJ morphology in other muscle groups. NMJ-morph analysis revealed comparable NMJ morphology across RA and 4 lower limb muscles (Supplemental Figure 2).

Quantitative NMJ-morph analyses confirmed that there were no significant differences in any aspect of NMJ morphology in RA across the 3 patient groups (Figure 3 and Supplemental Table 2). Crucially, and in stark contrast to predictions based on mechanistic animal models, there was no evidence of denervation (defined by percentage overlap [Figure 3C] between nerve terminal and endplate), demonstrating that this is not a major feature of pathogenesis in cachectic patients. Similarly, there was no evidence for increased NMJ fragmentation (Figure 3D), a classical feature of NMJ pathology found in animal models of neurodegeneration (7–9) and cardiac cachexia (33).

Alongside analyses of denervation and NMJ fragmentation, our NMJ-morph analysis confirmed no statistically significant changes in any of the other morphological variables investigated (Figure 3 and Supplemental Table 2); similar axon diameters (Figure 3A) (approximately 1 μm) were observed across all 3 groups, with no evidence of axonal swelling, neurofilament accumulation, or polyneuronal innervation (indicative of denervation/reinnervation processes). Thus, neither gross nor subtle perturbations at the NMJ were observed in cachectic patients. However, the relative contribution of muscle regeneration and myopathic changes still requires definitive demonstration in human cachexia patients.

It should be noted that cachectic patients in the current study represent the more extreme end of the clinical diagnostic definition criteria, having both weight loss and low CT muscularity. However, our study only enrolled patients who were eligible for surgery with potentially curative intent. It is not possible, therefore, to draw conclusions concerning a possible late disruption of the NMJ in palliative cancer patients with refractory cachexia and severe functional impairment.

While RA proved to be an excellent muscle for the current study, our findings differed from those observed in human age-related sarcopenia and in animal models of muscle wasting, both situations in which weight-bearing muscles are usually assessed experimentally. It remains possible therefore that skeletal muscle with different functional and/or biochemical properties may respond differently in cachexia. Equally, the observed differences between human patients and animal models may reflect anticipated differences in cachexia pathophysiology between the two. Human cachexia is proposed to be a multifactorial condition in which diverse drivers of muscle wasting all contribute to varying degrees in individual patients and tumor types (15). In comparison, in vivo tumor-bearing models may demonstrate accelerated wasting, which lacks the complexity and heterogeneity of the human condition. This supposition is supported by previous studies that have demonstrated little overlap in gene expression profiles between muscle biopsies from human cancer patients and equivalent animal models (34).

All patients received intravenous atracurium besilate during anesthesia, which competitively displaces acetylcholine from its receptors. Its half-life is 17 to 21 minutes, but whether it has longer lasting effects on the form or function of the NMJ is not known (35). Importantly therefore, samples from the current study were compared with lower limb samples from patients who had received spinal anesthesia only. No differences were observed, suggesting that the choice of anesthetic was unlikely to have had any significant impact on the morphology of the NMJ.

In summary, we report that the human NMJ retains full structural integrity in both cachexia and weight-stable cancer. This suggests that denervation of skeletal muscle and/or NMJ disruption are not major drivers of disease pathogenesis in cancer cachexia and that cancer cachexia represents a unique neuromuscular condition that needs to be differentiated from related conditions, including age-related sarcopenia. This observation supports the hypothesis that intrinsic changes within skeletal muscle, independent of any changes in motor neurons, represent the primary locus of pathology in cachexia. Since the NMJ remains intact in patients with cancer cachexia, promotion of muscle hypertrophy using exercise and neural stimulation should remain a viable therapeutic intervention for future clinical trials.

## Methods

### Patient recruitment.

Patients with a confirmed diagnosis of GI cancer suitable for surgical resection with curative intent were recruited from the regional multidisciplinary team meeting (*n* = 20; Supplemental Table 1) and included patients with both cancer cachexia (*n* = 10) and weight-stable disease (*n* = 10) based on consensus definition (3). Suitable age-matched control patients undergoing a range of elective abdominal procedures (e.g., repair of aortic aneurysm or donor nephrectomy) were also recruited (*n* = 10; Supplemental Table 1). All patients were at least 18 years of age and provided written, informed consent (inclusion criteria). Patients with a previous history of malignancy were not eligible for the control cohort (exclusion criteria).

### Body composition analysis.

See Supplemental Methods.

### Tissue sampling.

Tissue sampling was performed under general anesthesia at the start of the surgical procedure. If patients underwent neoadjuvant chemotherapy, surgery was performed 4 to 6 weeks following cessation of treatment. Biopsies of RA muscle were obtained following initial opening of the abdomen. RA is a well-characterized tissue for the study of human cancer cachexia, for which the results of previous studies have been reviewed recently (26). Evident changes of wasting, including fiber atrophy, have been robustly and repeatedly observed in several studies (32). RA has an “in-series” architecture, with motor endplate bands located throughout its length (36). To ensure successful sampling, a longitudinal strip of muscle lying between 2 contiguous tendinous intersections was obtained using sharp dissection (approximately 0.5 × 0.5 × 2.0 cm). Samples were immediately fixed in 4% paraformaldehyde (PFA) for approximately 2 hours, followed by washing and storage in 1× PBS.

### Tissue processing and NMJ immunohistochemistry.

See Supplemental Methods.

### Confocal imaging & NMJ-morph analysis.

See Supplemental Methods.

### Statistical analysis.

Statistical analyses were performed using GraphPad Prism 6 software. Results are expressed as mean (± SEM). Group comparison of normally distributed data (based on D’Agostino-Pearson test) was performed by 1-way ANOVA and Tukey’s post hoc test; nonparametric data were analyzed by Kruskal-Wallis test and Dunn’s post hoc test. *P* < 0.05 was considered significant.

### Study approval.

Ethics approval for tissue sampling was granted by the National Health Service Lothian Ethics Committee (IRAS 190214) in accordance with the Helsinki Declaration. All patients were at least 18 years of age and provided written informed consent.

## Author contributions

All authors designed the experiments and drafted the manuscript. IB, JM, RJES, RAJ, and THG performed the studies and analyzed the data. Authorship order was agreed on according to the time and effort committed to the project.

## Supplementary Material

Supplemental data

## Version 1 12/03/2019

In-Press Preview

## Version 2 02/10/2020

Electronic publication

## Version 3 03/02/2020

Print issue publication
