# Supplementary material for: Neuromuscular junctions are stable in patients with cancer cachexia
Source: J Clin Invest. 2020 Feb 10;130(3):1461–5. doi: 10.1172/JCI128411 (PMC7269586; doi:10.1172/JCI128411)
Supplement: Supplemental data [file jci-130-128411-s206.pdf]

**Supplemental Information for Boehm et al.**

**Supplementary Figure S1**

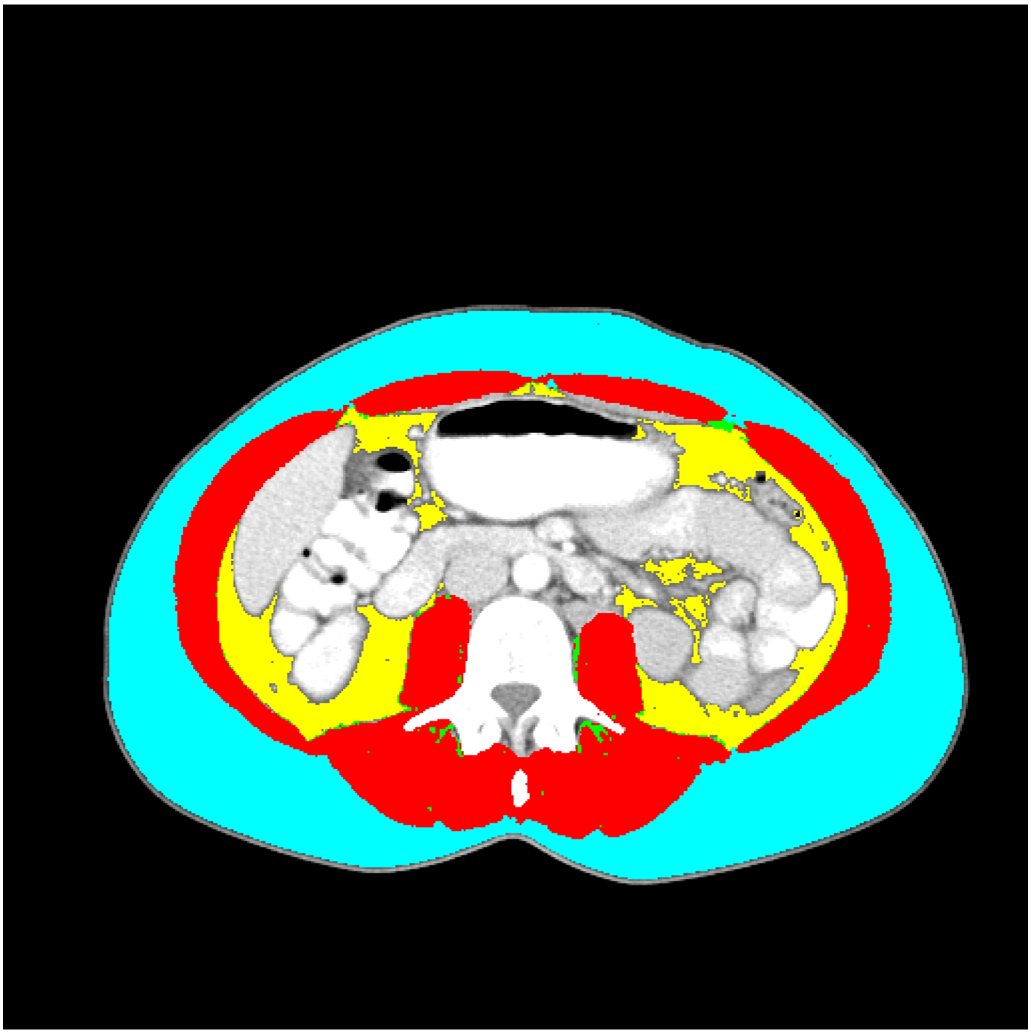

**Figure S1. Cross sectional CT image at L3 showing demarcation of skeletal muscle and adipose tissue using Slice-o-matic and ABACS software.**

Red = Skeletal Muscle, Green = Intramuscular fat, Yellow = Visceral adipose tissue, Blue = Subcutaneous adipose tissue

**Supplementary Figure S2**

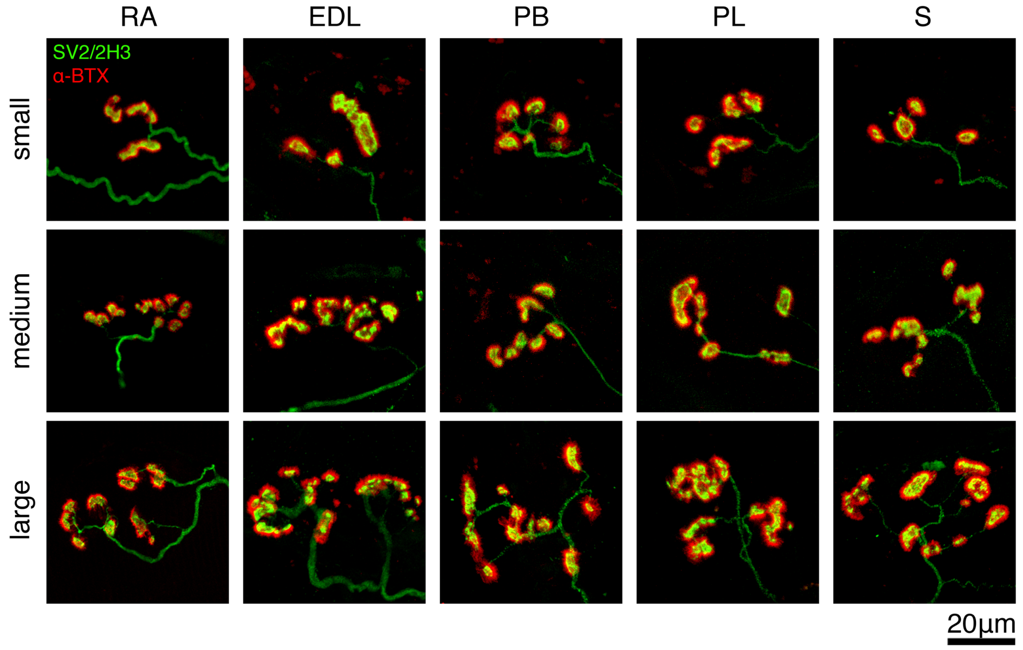


**Figure S2. Comparative anatomy of human NMJs in *rectus abdominis* (RA), *extensor digitorum longus* (EDL), *peroneus brevis* (PB), *peroneus longus* (PL) and *soleus* (S)**

No major differences in NMJ morphology were observed between lower limb muscles *extensor digitorum longus* (EDL, n = 10 patients), *peroneus brevis* (PB, n = 10 patients), *peroneus longus* (PL, n = 10 patients), *soleus* (S, n = 10 patients) and *rectus abdominis* (RA, n = 6 patients). The lower limb data of 10 patients from the previously published data set in Jones et al 2017 was chosen for its completeness of pre- and post-synaptic parameters. SV2/2H3 = synaptic vesicle 2 and neurofilament (presynapse, green); α-BTX = α-bungarotoxin (acetylecholine receptors, red). Scale bar = 20 µm. Box and whisker plots show the mean (± SEM) as a '+' over the median.

## Supplementary Table S1

| Patient Group (n = 10) | Ctrl | Weight Stable | Cachexia | F (DFn, DFd) or Kruskal Wallis statistic | P | Mean Difference Ctrl vs Weight Stable | Mean Difference Ctrl vs Cachexia | Mean Difference Weight Stable vs Cachexia |
|---|---|---|---|---|---|---|---|---|
| **Male:Female** | 9:1 | 7:3 | 8:2 | / | / | / | / | / |
| **Age (years)** | 64 ± 2.89 (min 49, max 81) | 66 ± 3.89 (min 40, max 83) | 68 ± 3.00 (min 49, max 78) | F (2, 27) = 0.3887 | 0.682 | -1.90 | -4.10 | -2.20 |
| **BMI** | 28.68 ± 1.32 (min 20.0, max 36.2) | 27.00 ± 1.78 (min 22.4, max 41.1) | 25.69 ± 1.13 (min 20.4, max 31.8) | F (2, 27) = 1.096 | 0.349 | 1.69 | 3.00 | 1.31 |
| **% weight loss** | 0 % ± 0 % (min 0 %, max 0 %) | 0.89 % ± 0.5 % (min 0 %, max 4.38 %) | 7.99 % ± 1.65 % (min 2.70 %, max 19.1 %) | 22.71 | < 0.0001 **** | -3.400 % | -16.10 % **** | -12.70 % ** |
| **SMI** | 52.0 ± 2.08 (min 38.5, max 59.6) | 56.9 ± 4.0 (min 43.7, max 78.5) | 40.3 ± 1.75 (min 30.6, max 52.3) | 12.98 | 0.0015 ** | -2.100 | 11.10 * | 13.20 ** |
| **VATI** | 64.4 ± 9.85 (min 15.8, max 111.6) | 53.0 ± 16.6 (min 6.73, max 188.4) | 73.7 ± 11.5 (min 33.5, max 134.0) | F (2, 27) = 0.644 | 0.5329 | 11.39 | -9.342 | -20.73 |
| **SATI** | 67.3 ± 14.0 (min 2.58, max 153.2) | 60.3 ± 11.3 (min 18.32, max 132.3) | 55.7 ± 4.53 (min 39.1, max 82.1) | 0.281 | 0.8688 | 1.900 | 1.700 | -0.2000 |
| **Cancer type:<br>Oesophageal ACC<br>Oesophageal SCC<br>Oesophageal undifferentiated<br>Gastric ACC<br>Colonic ACC** | / | 4<br>1<br>0<br>4<br>1 | 7<br>1<br>1<br>1<br>0 | / | / | / | / | / |
| **ECOG** | / | 0 (min 0, max 1) | 0 (min 0, max 1) | / | / | / | / | / |

**Table S1. Demographics of GI cancer patients recruited for NMJ analysis.** All data are mean and standard error of the mean (SEM), except for ECOG performance score, which is represented as median with range. Statistical significance for age and BMI was determined using a one-way ANOVA with a Tukey's post-hoc test. Statistical significance for % weight loss, SMI and SATI was determined using a Kruskall-Wallis test with a Dunn's post-hoc test. BMI = body mass index; SMI = skeletal muscle index VATI = visceral adipose tissue index; SATI = subcutaneous adipose tissue index; ECOG = Eastern Cooperative Oncology Group performance status, ACC = adenocarcinoma, SCC = squamous cell carcinoma.

## Supplementary Table S2

| | Ctrl n = 10 387 NMJs | | | Weight Stable n = 10 386 NMJs | | | Cachexia n = 10 392 NMJs | | | F (DFn, DFd) or Kruskal Wallis statistic | P | Mean Difference Ctrl vs Weight Stable | Mean Difference Ctrl vs Cachexia | Mean Difference Weight Stable vs Cachexia |
|---|---|---|---|---|---|---|---|---|---|---|---|---|---|---|
| **core variables** | | | | | | | | | | | | | | |
| pre-synaptic | | | | | | | | | | | | | | |
| ***1)*** *Nerve Terminal Area (μm$^2$)* | 76.53 | ± | 6.84 | 73.70 | ± | 11.08 | 74.86 | ± | 10.02 | F (2, 27) = 0.023 | 0.978 | 2.832 | 1.669 | -1.163 |
| ***2)*** *Nerve Terminal Perimeter (μm)* | 143.22 | ± | 9.88 | 136.16 | ± | 13.49 | 134.90 | ± | 11.67 | F (2, 27) = 0.145 | 0.866 | 7.061 | 8.318 | 1.257 |
| ***3)*** *Number of Terminal Branches* | 37.88 | ± | 3.21 | 37.01 | ± | 2.56 | 37.42 | ± | 2.47 | 0.008 | 0.996 | -0.250 | -0.350 | -0.100 |
| ***4)*** *Number of Branch Points* | 17.29 | ± | 3.01 | 17.39 | ± | 1.98 | 17.66 | ± | 1.94 | F (2, 27) = 0.007 | 0.994 | -0.097 | -0.097 | -0.272 |
| ***5)*** *Total Length of Branches (μm)* | 64.16 | ± | 6.08 | 61.25 | ± | 6.98 | 60.02 | ± | 5.99 | F (2, 27) = 0.112 | 0.895 | 2.916 | 4.148 | 1.232 |
| post-synaptic | | | | | | | | | | | | | | |
| ***6)*** *AChR Area (μm$^2$)* | 129.92 | ± | 9.66 | 121.98 | ± | 14.53 | 124.80 | ± | 15.01 | F (2, 27) = 0.092 | 0.913 | 7.935 | 5.117 | -2.818 |
| ***7)*** *AChR Perimeter (μm)* | 150.94 | ± | 9.48 | 154.28 | ± | 15.64 | 153.86 | ± | 18.94 | F (2, 27) = 0.014 | 0.986 | -3.338 | -2.922 | 0.416 |
| ***8)*** *Endplate Area (μm$^2$)* | 241.02 | ± | 21.06 | 228.18 | ± | 29.65 | 221.47 | ± | 29.81 | F (2, 27) = 0.134 | 0.875 | 12.840 | 19.550 | 6.711 |
| ***9)*** *Endplate Perimeter (μm)* | 77.84 | ± | 4.28 | 77.42 | ± | 6.36 | 77.32 | ± | 6.67 | F (2, 27) = 0.002 | 0.998 | 0.421 | 0.520 | 0.099 |
| ***10)*** *Endplate Diameter (μm)* | 27.30 | ± | 1.32 | 28.39 | ± | 2.35 | 27.99 | ± | 2.25 | F (2, 27) = 0.074 | 0.929 | -1.093 | -0.690 | 0.403 |
| ***11)*** *Number of AChR Clusters* | 4.15 | ± | 0.28 | 4.12 | ± | 0.42 | 4.28 | ± | 0.46 | F (2, 27) = 0.045 | 0.956 | 0.023 | -0.131 | -0.154 |
| **derived variables** | | | | | | | | | | | | | | |
| pre-synaptic | | | | | | | | | | | | | | |
| ***12)*** *Average Length of Branches (μm)* | 1.91 | ± | 0.11 | 1.78 | ± | 0.15 | 1.78 | ± | 0.10 | F (2, 27) = 0.415 | 0.665 | 0.132 | 0.135 | 0.003 |
| ***13)*** *Complexity* | 4.35 | ± | 0.15 | 4.34 | ± | 0.12 | 4.37 | ± | 0.12 | F (2, 27) = 0.011 | 0.989 | 0.011 | -0.016 | -0.027 |
| post-synaptic | | | | | | | | | | | | | | |
| ***14)*** *Average Area of AChR Clusters (μm$^2$)* | 42.32 | ± | 4.65 | 37.65 | ± | 3.64 | 38.87 | ± | 2.83 | F (2, 27) = 0.410 | 0.667 | 4.669 | 3.448 | -1.221 |
| ***15)*** *Fragmentation* | 0.65 | ± | 0.032 | 0.63 | ± | 0.04 | 0.62 | ± | 0.03 | F (2, 27) = 0.132 | 0.877 | 0.016 | 0.025 | 0.009 |
| ***16)*** *Compactness (%)* | 56.08 | ± | 1.32 | 55.97 | ± | 1.79 | 59.01 | ± | 1.13 | F (2, 27) = 1.439 | 0.255 | 0.108 | -2.939 | -3.047 |
| ***17)*** *Overlap (%)* | 46.01 | ± | 0.9986 | 46.84 | ± | 2.13 | 48.18 | ± | 2.54 | F (2, 27) = 0.301 | 0.742 | -0.836 | -2.173 | -1.337 |
| ***18)*** *Area of Synaptic Contact (μm$^2$)* | 58.94 | ± | 4.56 | 57.02 | ± | 8.30 | 58.07 | ± | 7.36 | F (2, 27) = 0.019 | 0.981 | 1.919 | 0.866 | -1.053 |
| **associated nerve & muscle variables** | | | | | | | | | | | | | | |
| ***19)*** *Axon Diameter (μm)* | 1.14 | ± | 0.06 | 1.03 | ± | 0.06 | 1.07 | ± | 0.06 | 2.178 | 0.337 | 5.800 | 3.200 | -2.600 |
| ***20)*** *Muscle Fibre Diameter (μm)* | 79.82 | ± | 1.25 | 79.11 | ± | 1.02 | 70.44 | ± | 1.06 | F (2, 1132) = 22.350 | < 0.0001 | 0.714 | 9.383 **** | 8.669 **** |
| ***21)*** *Number of Axonal Inputs* | 1.08 | ± | 0.03 | 1.08 | ± | 0.04 | 1.03 | ± | 0.10 | 0.239 | 0.888 | 1.650 | 0.000 | -1.650 |

**Table S2. Overview of complete NMJ-morph data.**

The mean values ± SEM are listed for each patient group, representing the mean of the entire analysed NMJ data set. Statistical significance from a one-way ANOVA paired with a Tukey's post-hoc test. Statistical significance of average area of AChR clusters and fragmentation determined via Kruskal Wallis non-parametric test, followed by a Dunn's post-hoc test.

**Supplementary Methods**

*Body composition analysis*

Skeletal muscle index (SMI) was calculated either from routine staging CT scans performed prior to surgical intervention, or the post chemotherapy re-staging scan if the patients underwent neoadjuvant chemotherapy (n = 5 in the cachectic group and n = 3 in the weight stable group). Digital CT images obtained with a spiral CT scanner were analysed using Slice-O-matic and ABACS software (Supplementary Figure 1). SMI was derived from measurements of muscle cross-sectional area normalized to body stature ($cm^2/m^2$) at the level of the 3rd lumbar vertebra (L3) (Supplementary Figure 1). SMI cut-offs for low SMI were obtained from previously published reference data (30). Cachexia was defined according to standard consensus (>2% weight loss and low muscularity (3) as this definition has been shown to demonstrate histological muscle wasting in previous studies (32).

*Tissue processing and NMJ immunohistochemistry*

Teased muscle fibre preparations were immunohistochemically labelled for NMJ analysis as previously described (24) to visualise key pre-synaptic proteins (the synaptic vesicle protein SV2 and the neurofilament protein 2H3), with post-synaptic acetylcholine receptors (AChRs) labelled using tetramethylrhodamine-conjugated alpha-bungarotoxin. Optimal labelling was achieved with incubation in primary antibodies for 3 nights and secondary antibodies for 1 night. Samples were whole-mounted in Mowiol and stored at -20$^{o}$C prior to imaging.

Primary antibodies: mouse monoclonal SV2 and 2H3 (1:50; Developmental Studies Hybridoma Bank, University of Iowa). Secondary antibodies: AlexaFluor-488-conjugated donkey anti-mouse IgG (H+L) antibody (1:400; A21202, Life Technologies). Tetramethylrhodamine-conjugated alpha-bungarotoxin (2 µg/mL; BTIU00012, VWR International).

SV2 and 2H3 antibodies, developed by Buckley, K.M. and Jessell, T.M. / Dodd, J. respectively, were obtained from the Developmental Studies Hybridoma Bank, created by the NICHD of the NIH and maintained at The University of Iowa, Department of Biology, Iowa City, IA 52242.

*Confocal imaging & NMJ-morph analysis*

Confocal images of individual en face NMJs and their pre-terminal axons were acquired on a Zeiss LSM 710 or Nikon A1R confocal laser-scanning microscope, using standardised imaging approaches (24,25). In addition, individual muscle fibre diameter measurements were obtained on an Olympus IX71 microscope and Hamamatsu C4742-95 camera with Openlab Improvision software (Jones et al. 2016; Jones et al. 2017). All images were then analysed according to a standardised workflow ('NMJ-morph'), as previously described (24,25). NMJ-morph data output generates 21 separate morphological descriptors for each NMJ (see Supplementary Table 2), including pre- and post-synaptic variables (e.g. nerve terminal area, motor endplate area, etc.), derived variables (e.g. nerve terminal complexity, motor endplate fragmentation, etc.) and related nerve and muscle measurements (including motor axon diameter and muscle fibre diameter). As per NMJ-morph guidelines, a total of 40 NMJs were analysed (where possible) for each individual patient/muscle sample (N = 30 patients) giving a complete dataset of n = 1,165 NMJs.
